# Study the effect of *Enterobacter cloacae* on the gene expression, productivity, and quality traits of *Curcuma longa* L. Plant

**DOI:** 10.3389/fpls.2024.1393198

**Published:** 2024-08-02

**Authors:** Hind Salih Alrajeh, Fadia El Sherif

**Affiliations:** ^1^ Department of Biological Sciences, College of Science, King Faisal University, Al Ahsa, Saudi Arabia; ^2^ Department of Horticulture, Faculty of Agriculture, Suez Canal University, Ismalia, Egypt

**Keywords:** Turmeric, PGPB, RT-PCR, rhizome, NPK, HPLC

## Abstract

Overuse of artificial chemical fertilizers could be detrimental to the environment. Utilizing beneficial microorganisms as biofertilizers is a sustainable technique that promotes soil health, crop yield, and ecosystem preservation. *Curcuma longa* L. is utilized as a medication since it has its antibacterial, anti-microbial, and anti-tumor characteristics, which reduce inflammation and hasten wound healing. The effect of *E. cloacae* strain MSR1, which is common in the roots of alfalfa grown in the Al-Ahsaa region, on *C. longa* plants is being investigated. *C. longa* rhizomes were planted under greenhouse conditions after being submerged in a solution of *E. cloacae* strain MSR1 (OD 500) or water treatment as a control for 12 hours. After 240 days of growing, ten randomly selected plants from each treatment were collected, and the vegetative growth and yield metrics were assessed. To investigate how *E. cloacae* influences *C. longa* production and chemical composition (photosynthetic pigment, nitrogen, phosphorus, potassium, and curcuminoid), measurements were conducted as well as genes diketide-CoA and curcumin synthases genes. Our research showed that *C. longa's* growth and yield were favorably impacted by *E. cloacae.* Significant increases in the related plants' chlorophyll a,b, carotenoid, nitrogen, phosphorus, and potassium levels were likewise a reflection of the enhanced effects shown in the growth and yield parameters. Treatment with *E. cloacae* raised the curcuminoid's three sub-components' compositions to varying degrees: bisdemethoxycurcumin, demethoxycurcumin, and curcumin. Comparing *E. cloacae* treated plants to the control, high expression levels of the genes diketide-CoA and curcumin synthase-1, -2, and 3 were also found. The treatment of *E. cloacae* is a good biostimulant candidate for boosting growth and yield as well as raising the medicinal qualities of C. longa, according to the overall results.

## Introduction

The excess use of fertilizers made from synthetic chemicals could be detrimental to the environment. Consequently, suggestions have been made to replace chemical fertilizers entirely or in part with alternative sources, particularly biofertilizers (Bisht and Singh Chauhan, 2021). These biofertilizers are affordable and sustainable. The use of biofertilization techniques to produce organic food has emerged as a key technological advance for safeguarding public health, cropland, and the environment. Food production methods have multiplied, particularly for crops such as turmeric that have high commercial value and promising medical applications (Singh et al., 2016).

Turmeric, scientifically known as *Curcuma longa* L., is a perennial herbaceous plant belonging to the Zingiberaceae family (Sotiboldieva and Mahkamov, 2020). Rhizomes are commonly consumed plant parts that are rich in volatile oil compounds (e.g., mono- and sesquiterpenoids) and non-volatile curcuminoids, which have bioactive properties (Sharifi-Rad et al., 2020; Klopchevska et al., 2022). The bioactive compound in turmeric responsible for its health benefits is called curcumin. Curcumin has antioxidant, anti-inflammatory, neuroprotective, and anticancer properties (Sharifi-Rad et al., 2020; Aminnezhad et al., 2023). Dipeptide-CoA synthase (*DCS*) and curcumin synthases 1, 2, and 3 are the genes that mediate curcuminoid metabolism in *C. longa* (Chakraborty et al., 2021; Katsuyama et al., 2009a; Katsuyama et al., 2009b; Sandeep et al., 2017).

The use of beneficial microorganisms as biofertilizers is a sustainable technique that promotes soil health and crop yield. Beneficial microorganisms employ two distinct ways that either directly or indirectly enhance plant productivity: phytopathogen suppression and plant growth promotion (PGP) (Koskey et al., 2021). *Enterobacter cloacae* belongs to the Enterobacteriaceae family and is a Gram-negative, short rod bacterium (García-González et al., 2018).

*E. cloacae* strains that exhibit numerous growth-promoting properties, such as phosphate solubilization, nitrogen fixation, phytohormone production, and exopolysaccharide production, have been found to be plant growth promoters (Gupta et al., 2022). *E. cloacae* has been essential in establishing and maintaining soil fertility, which increases the development and yield of several agricultural crops. These qualities are due to the various traits of this bacterium that encourage plant growth, including its solubility in phosphate, its ability to produce phytohormones such as acetoin and phosphate, and its ability to produce bioactive compounds (Khalifa et al., 2016; García-González et al., 2018; Al Dayel and El Sherif, 2021; Sritongon et al., 2023).

The objective of this study was to comprehensively examine and assess the effects of treatment with *E. cloacae* on the plant growth, yield, and bioactive substances of *C. longa*. The curcuminoid gene expression patterns in the rhizomes were thoroughly investigated to improve our understanding of the underlying molecular mechanisms and obtain a deeper understanding of the molecular mechanisms involved. The effect of the *E. cloacae* strain MSR1, which is present in the roots of alfalfa growing in the Al-Ahsaa region, on *C. longa* plants is studied for the first time in this paper.

## Materials and methods

### Turmeric cultivation and bacterial treatment

The turmeric rhizomes (40 g) (Narendhiran et al., 2024) were submerged in a solution of the *E. cloacae* strain MSR1 (Khalifa et al., 2016) at 25°C for 12 h, as provided by the Microbiology Laboratory at the Department of Biological Science, Faculty of Science, King Faisal University. The optical density of the solution was measured at OD_500_)the concentration used in the experiment was 10^6^ microbial cells per 1 ml fluid), while the control method involved soaking in distilled water. On April 1, 2022, the turmeric rhizomes were planted in sand-filled germination trays within the greenhouse of the King Faisal University Agriculture and Veterinary Research and Training Centre, King Faisal University (25.266184323290517 and 49.695981580002844). After 1 month, the seedlings (5 cm long and with three leaf pairs) were cultured into 20-cm-wide and 15-cm-deep plastic pots filled with 4.5 kg of sand soil per pot (one plant per pot).

The percentage of rhizomes that germinated was recorded. The tests utilized a completely randomized block design with 15 replicates (pots), two treatment groups of *E. cloacae*, and distilled water as the control (Al Dayel and El Sherif, 2021). Groundwater irrigated each plant, as needed (**Supplementary Table S1**). The soil and irrigation water components were identified using the method of Buurman et al. (1996) (**Supplementary Tables S1, S2**). After 240 days of cultivation, 10 randomly chosen plants from each treatment were collected. The following measurements were made: plant height (in centimeters); rhizome diameter (in millimeters); dry weight of the roots, leaves, and rhizomes per plant (in grams); and the number of roots, leaves, and rhizomes per plant (*n*).

### Chemical analysis

#### Photosynthetic pigment measurement

A random sample of four 240-day-old turmeric plants was selected. The photosynthetic pigment content was measured in the third leaf from the apex of each plant. Chlorophylls a and b, as well as the carotenoids, were extracted and computed according to the methods described in House et al. (2019).

#### Mineral composition

After collection (240 days from planting in the field) from various treatments, the leaves from the turmeric plant were dried at 60°C for 48 h. Subsequently, the leaves were degraded with sulfuric acid following Piper’s description in 1942 (Piper, 2019). The modified micro-Kjeldahl technique introduced by Jackson in 1967 (Nirere et al., 2021) was employed to determine the nitrogen content.

Similarly, to determine the phosphorus level, calorimetry, as suggested by Murphy and Riley in 1962 (Amm et al., 2023), was performed, while the potassium concentration was determined through atomic absorption flame photometry, as proposed by Mazumdar and Majumder in 2003 (Banerjee and Prasad, 2020). Finally, the soil samples were collected and assessed at the conclusion of the study based on the water and soil studies conducted by Page in 1982 (Bottomley et al., 2020).

### HPLC analysis of the curcuminoid content in *C. longa* rhizome

This study used air-dried powdered *C. longa* rhizome from three plants randomly selected for each treatment of *E. cloacae* and the control. The concentrations of curcumin, bisdemethoxycurcumin, and demethoxycurcumin were determined with a Waters 2690 Alliance HPLC system. The system was equipped with a C18 Inertsil column (4.6 mm × 250 mm, 5 m) and a Waters 996 photodiode array detector. The analysis was conducted following the guidelines outlined by Field (El Sherif et al., 2022).

### Real-time RT-PCR analysis of curcuminoid gene expression

Real-time reverse transcriptase polymerase chain reaction (RT-PCR) was used to measure the transcript concentrations of the curcuminoid genes (*CURS1*, *CURS2*, *CURS3*, and *DCS*) in *C. longa* rhizomes. From each experimental group, four 240-day-old plants were chosen at random. The procedures were outlined as shown in **Supplementary Table S3** (Katsuyama et al., 2009a; Katsuyama et al., 2009b; El Sherif et al., 2022).

### Statistical evaluation

The experiment utilized a completely randomized block design, which was repeated 10 times. Data were compared using an independent-samples *t*-test in SPSS 21 software package (version 21.0; IBM Corp., Armonk, NY, USA).

## Results

### Influence of *E. cloacae* in enhancing plant production

**Table 1** displays the findings from the measurements of vegetative development. The results showed that treatment with *E. cloacae* led to a statistically considerable increase in the amount of leaves and roots, as well as the dry weight and length of *C. longa* roots, as compared with the control group. In contrast, compared with the control treatment, the administration of *E. cloacae* led to significant reductions in plant height.

**Table 1:** Influence of *Enterobacter cloacae* in enhancing the development of *Curcuma longa*.

Treatment	Plant height (cm)	No. of leaves (*n*)	No. of roots (*n*)	Root length (cm)	Weight of dried roots (g)	Weight of dried leaves (g)
Control	127^a^	9.67	30.67	15.67	1.73	17.77
*E. cloacae*	122	10.67^a^	34.0^a^	20.0^a^	2.13^a^	18.7^a^
Sig.	0.025	0.039	0.017	0.031	0.027	0.047

a *t*-test significant at *p* < 0.05.

**Figure 1:**
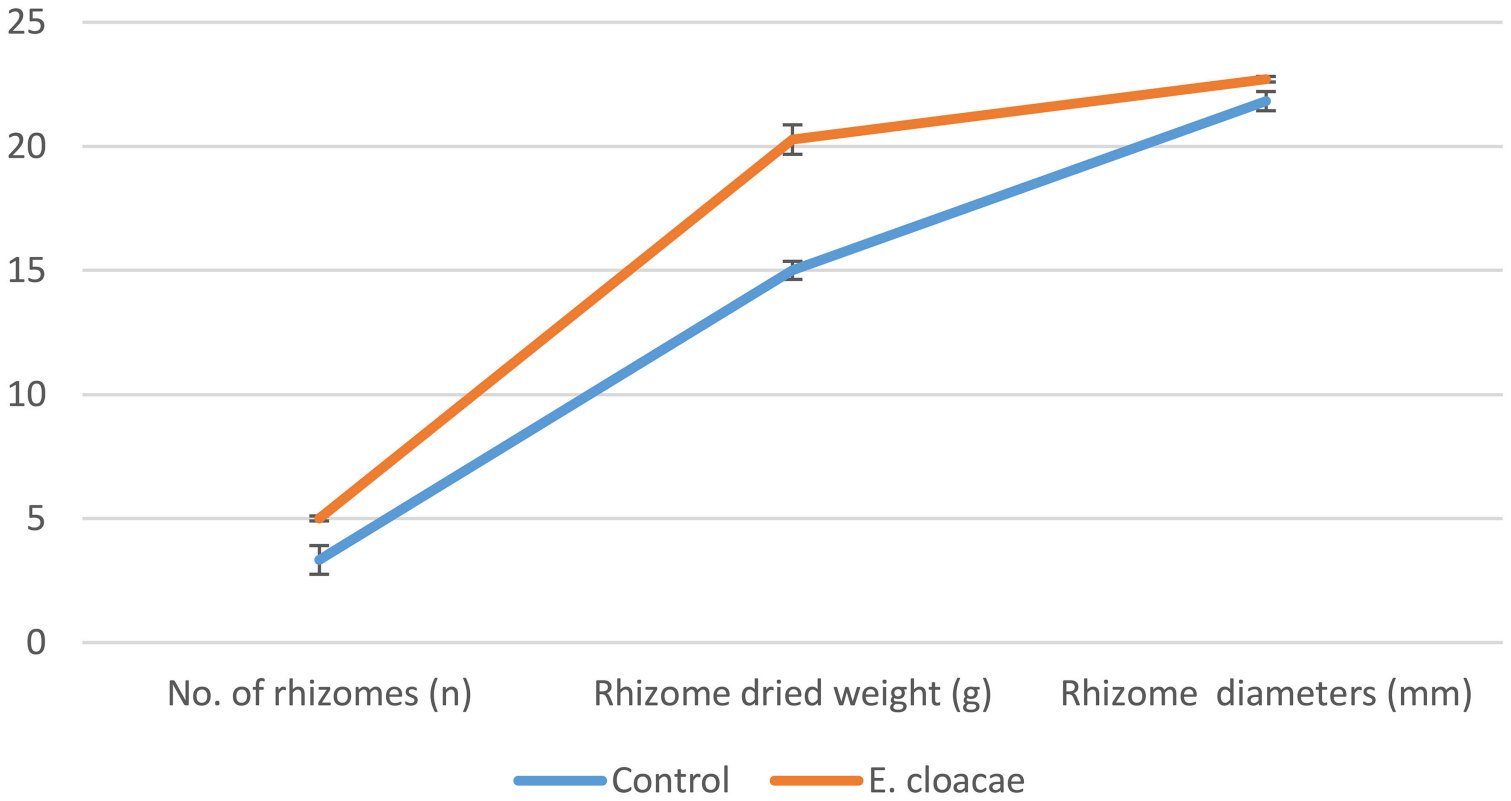
Effects of *Enterobacter cloacae* treatment on *Curcuma longa* yield.

As shown in **Figure 1**, treatment with *E. cloacae* significantly increased the amount, dry weight, and the diameter of rhizomes by approximately 1.5, 1.35, and 1.04 times, respectively, in contrast to the control treatment.

### Influence of *E. cloacae* on the photosynthetic pigment contents of *C. longa* leaves

According to **Table 2**, it appears that treatment of *C. longa* with *E. cloacae* resulted, in comparison to the control, in a notable increase in the quantity of carotenoid and chlorophylls a and b. As shown in the table, the increments were approximately 1.01-, 1.99-, and 1.25-fold, respectively.

**Table 2:** Effects of *Enterobacter cloacae* treatment on the photosynthetic pigments of *Curcuma longa* leaves.

Treatment	Chl a (mg/100 g FW)	Chl b (mg/100 g FW)	Carotenoids (mg/100 g FW)
Control	112.28	23.12	100.08
*E. cloacae*	113.43^a^	27.74^a^	125.47^a^
Sig.	0.031	0.051	0.277

Chla, chlorophyll a; Chlb, chlorophyll b; FW, fresh weight.

a *t*-test significant at *p* < 0.05.

### Influence of *E. cloacae* on the nitrogen, phosphorus, and potassium contents of *C. longa* leaves

**Table 3** presents the data on the impact of *E. cloacae* on the potassium, phosphorus, and nitrogen levels in the leaves of *C. longa*. Treatment with *E. cloacae* led to 1.09- and 1.14-fold increases in the phosphorus and nitrogen contents, and these increments were statistically significant. On the other hand, no significant increase in potassium content was observed.

**Table 3:** Influence of *Enterobacter cloacae* on the percentage of nitrogen, phosphorus, and potassium in *Curcuma longa* leaves.

Treatment	*k*%	P (ppm)	N (ppm)
Control	1.9434	0.1421	11.0973
*E. cloacae*	1.9474	0.1551^a^	12.656^a^
Sig.	0.073	0.012	0.041

a *t*-test significant at *p* < 0.05.

### Influence of *E. cloacae* on the curcuminoid content of *C. longa* rhizome

**Figure 2:**
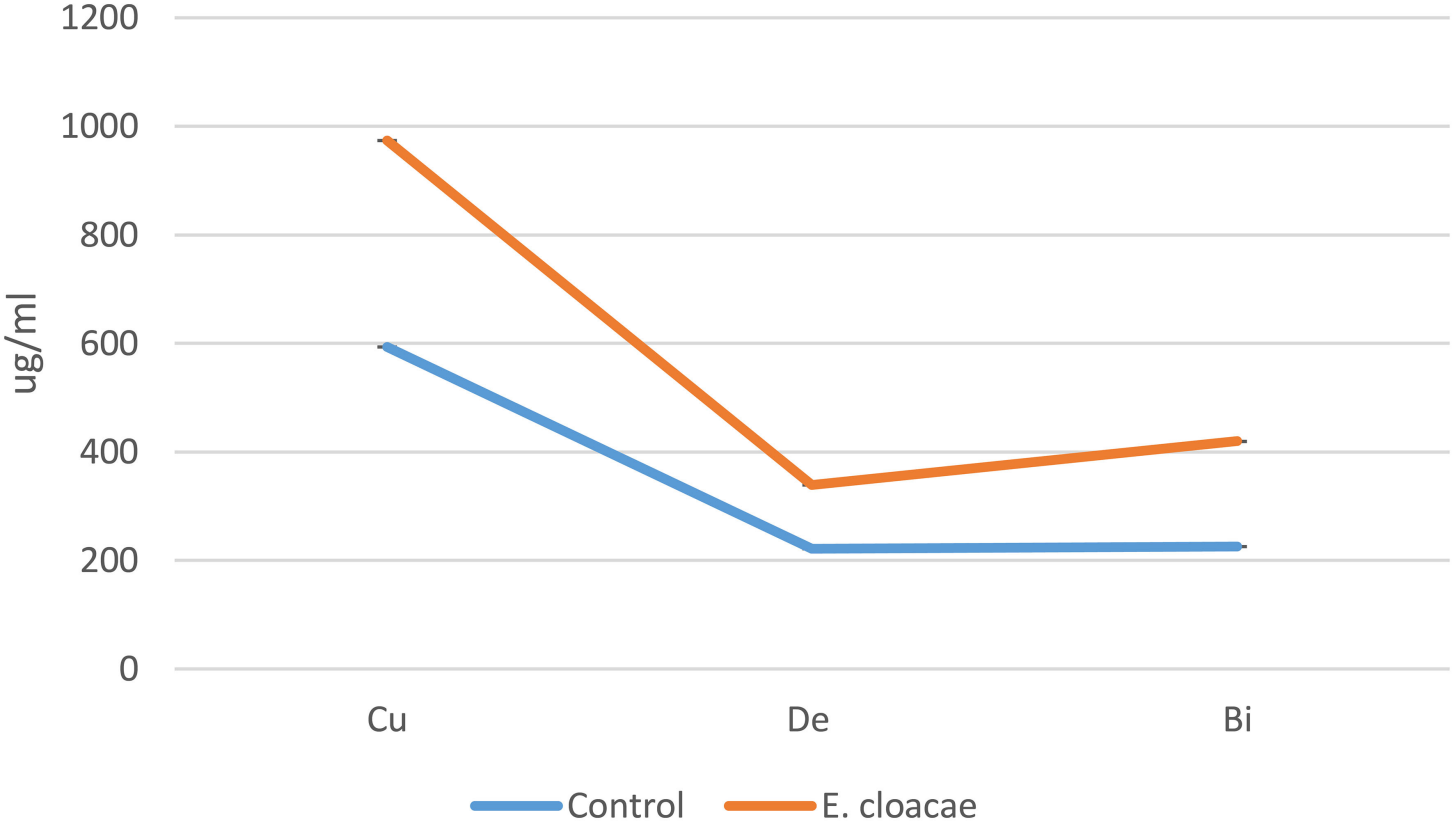
Effects of *Enterobacter cloacae* treatment on the curcuminoid accumulation (in micrograms per milliliter) of *Curcuma longa*.

**Figure 3:**
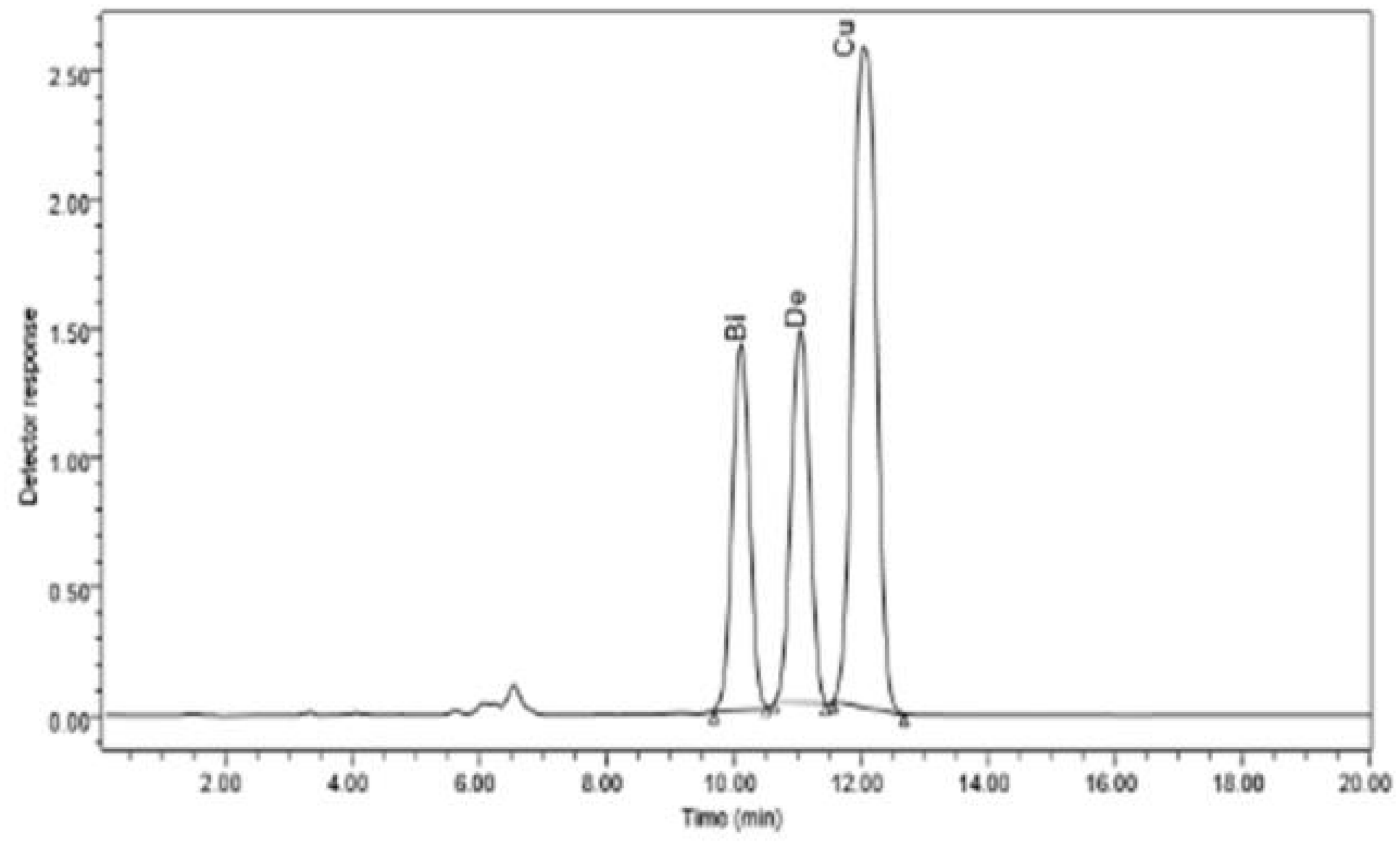
HPLC chromatogram of the organic extract of *Curcuma longa* after exposure to *Enterobacter cloacae*, which reveals the presence of curcumin, dimethoxycurcumin, and bisdemethoxycurcumin compounds.

The bioactive substances bisdemethoxycurcumin, demethoxycurcumin, and curcumin were much more prevalent in the methanolic extracts of *C. longa* rhizome after the application of *E. cloacae.* By using HPLC, it was possible to measure these increases, which were found to be 1.86-, 1.35-, and 1.64-fold higher than those of the control. These results are illustrated in **Figures 2** and **3**.

### Impact of *E. cloacae* on the expression of the curcuminoid synthase genes

**Figure 4:**
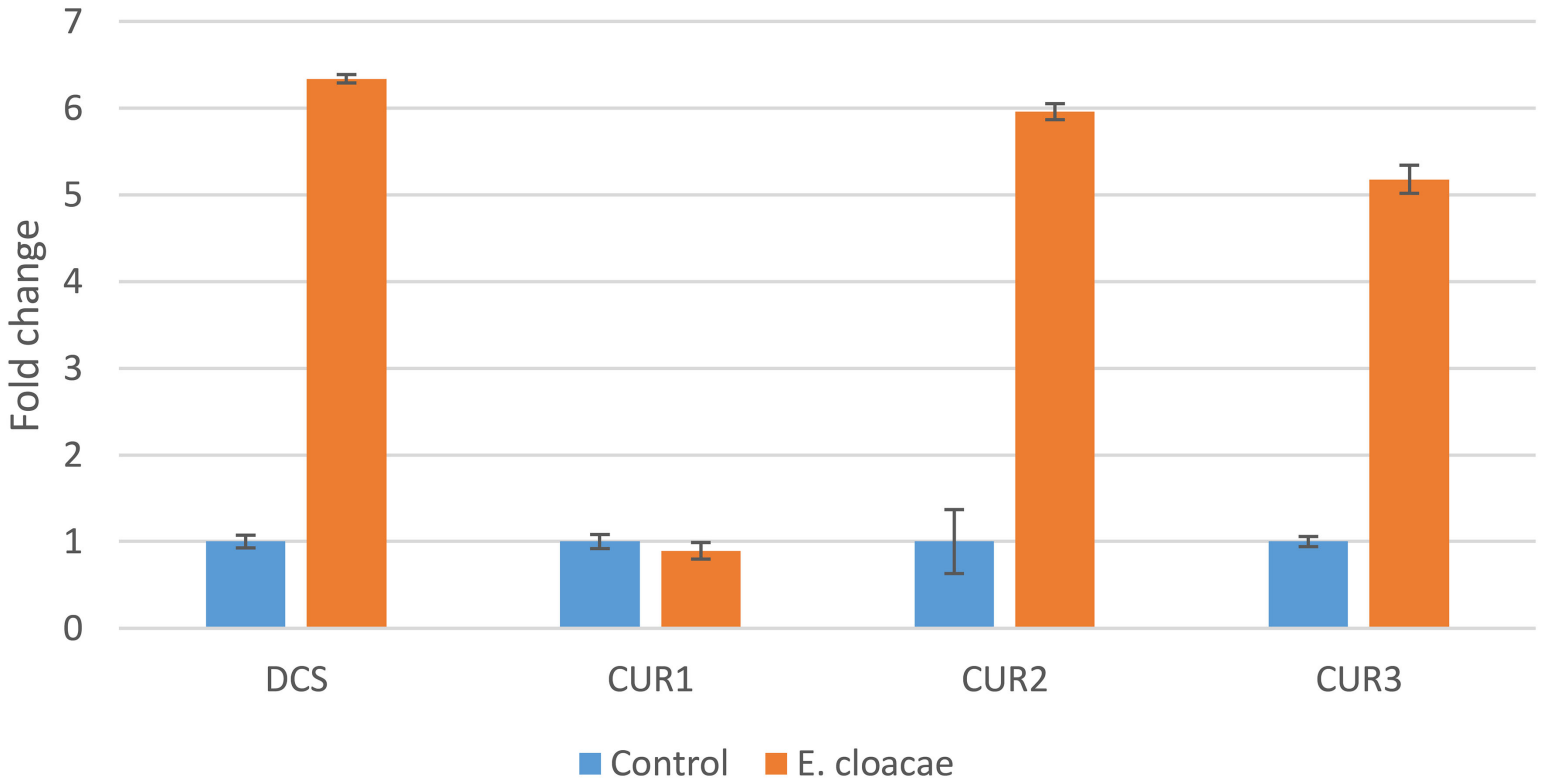
Expression of the curcuminoid synthase genes in *Curcuma longa* rhizome treated with *Enterobacter cloacae*. Actin was used as an internal reference gene for standardization.

The results of this study indicated that the genes *CURS2, CURS3*, and *DCS* were differentially upregulated by treatments with *E. cloacae*. The expression levels of *DCS*, *CURS*2, and *CURS3* were higher following *E. cloacae* treatment compared with the control (**Figure 4**). The use of *E. cloacae* increased the expression of *DCS*, *CURS*2, and *CURS*3 (6.3-, 5.9-, and 5.2-fold respectively) compared with the control (**Figure 4**). However, a gradual reduction in the expression of the *CUR1* gene was observed with *E. cloacae* application compared with the control treatment.

## Discussion

This study examined the impact of *E. cloacae* (as a plant growth-promoting bacterium, PGPB) on the growth and yield of *C. longa*. The results of this study contribute to the knowledge needed for the implementation of microorganisms that promote plant growth in an agricultural setting, such as curcuma plants.

It is worth noting that sand soil is considered deficient in mineral nutrients. The results presented in **Supplementary Table S1** validate this claim. According to Turan et al. (2017), *E. cloacae* stimulation of root proliferation could improve the capacity of the seedlings to sequester the limited mineral nutrients found in vermiculite, which may inadvertently promote shoot growth. Seedlings may also be more adept at sequestering minerals from vermiculite, which could sustain the growth observed in plants treated with PGPB. *E. cloacae* has high levels of nitrogen-fixing bacteria from the rhizosphere, which increases the N content in the soil (Chao et al., 2020). *E. cloacae* had the most favorable effect on the plant height, number of roots, dry weight of the leaves, and the contents of chlorophylls a and b, carotenoid, and N, P, and K in the leaves, which correlated with the highest rhizome number, rhizome dry weight, and rhizome diameter.The bacterium-associated roots of a plant are essential to its growth and development due to a variety of processes, including the availability of nutrients and the effect of the produced indoleacetic acid (IAA) on the root shape (Saikia et al., 2018; Chouhan et al., 2021; Shankar and Prasad, 2023). According to Overvoorde et al. (2010), with regard to root formation, this hormone primarily influences the length of the main root, the number of lateral roots, and the amount of root hairs. The effects of *E. cloacae* on plant development and productivity have been thoroughly documented (Ramakrishnan et al., 2023; Wang et al., 2023; Yue et al., 2023; Fan et al., 2024). *E. cloacae* caused an increase in the amount of photosynthetic pigments, increased the nutrient absorption, and increased the vegetative plant growth, and this enhanced rhizome output was sustained by the turmeric plant (Gupta et al., 2022; Chandarana and Amaresan, 2023; Shankar and Prasad, 2023; Sun et al., 2023). An increase in bioactive compounds resulting from the application of plant growth-promoting rhizome (PGPR) has been previously reported, including rutin and gallic acid in *Moringa oleifera* (Al Dayel and El Sherif, 2021), glucosinolate in *Brassica oleracea* (AbdElgawad et al., 2023), and acemannan in *Aloe vera* (Nikolaou et al., 2023).

The curcuminoid genes have been discovered as being involved in the production of curcuminoid in previous investigations (Katsuyama et al., 2009a; Katsuyama et al., 2009b). In this study, we found a simultaneous increase in the curcuminoid biosynthesis genes (*DCS*, *CURS2*, and *CURS3*) as demonstrated by RT-PCR and in the curcuminoids (curcumin, dimethoxycurcumin, and bisdemethoxycurcumin) as evaluated by HPLC. Similar results have been reported by El Sherif et al. (2022) and Khattab et al. (2023), suggesting that these genes play a role in modifying the levels of curcuminoids in *C. longa*.

## Conclusions

The utilization of *E. cloacae* as a biofertilizer has opened up numerous opportunities for agriculture. The findings of this study conclusively demonstrated that *E. cloacae* significantly exceeded the control treatments in terms of growth (root and shoot quantity, root length, and dry weight of leaves and roots) and yield (rhizome number, rhizome diameter, and root dry weight), as well as in the levels of photosynthetic pigments (chlorophylls a and b and carotenoids) and the contents of nitrogen, phosphorus, potassium, and curcuminoids (bisdemethoxycurcumin, demethoxycurcumin, and curcumin), all without the addition of external mineral nutrients, over the 240-day cultivation period. The unique influence of *E. cloacae* treatment on the expression of the genes *CURS2*, *CURS3*, and *DCS* also uncovered its potential application in plants. By utilizing *E. cloacae*, the complete potential of *C. longa* rhizome and other plants.

## Data availability statement

The original contributions presented in the study are included in the article/**Supplementary Material**, further inquiries can be directed.

## Author contributions

FE: Conceptualization, Data curation, Formal analysis, Methodology, Resources, Software, Writing – original draft, Writing – review & editing. HA: Funding acquisition, Investigation, Methodology, Writing – review & editing.
