# Corrigendum: Study the effect of *Enterobacter cloacae* on the gene expression, productivity, and quality traits of *Curcuma longa* L. Plant

**DOI:** 10.3389/fpls.2024.1493043

**Published:** 2024-10-17

**Authors:** Hind Salih Alrajeh, Fadia El Sherif

**Affiliations:** ^1^ Department of Biological Sciences, College of Science, King Faisal University, Al Ahsa, Saudi Arabia; ^2^ Department of Horticulture, Faculty of Agriculture, Suez Canal University, Ismalia, Egypt

**Keywords:** Turmeric, PGPB, RT-PCR, rhizome, NPK, HPLC

In the published article, there was an error with grant number in the **Funding** statement “grant number AN000634”. The correct **Funding** statement appears below.

“The Deanship of Scientific Research at King Faisal University is acknowledged by the authors for sponsoring this research project with grant number 5161.”

The authors apologize for this error and state that this does not change the scientific conclusions of the article in any way. The original article has been updated.

