# Supplementary material for: Study the effect of Enterobacter cloacae on the gene expression, productivity, and quality traits of Curcuma longa L. Plant
Source: Front Plant Sci. 2024 Aug 2;15:1393198. doi: 10.3389/fpls.2024.1393198 (PMC11327073; doi:10.3389/fpls.2024.1393198)
Supplement: Supplementary file 1 [file Table_1.docx]

**Supplementary Table 1: Chemical properties and compositions of the irrigation water.**

| **Salinity Level (ppm)** | **Cations (meq/L)** | | | | **Anions (meq/L)** | | | | **Sodium Adsorption Ratio (SAR)** |
| --- | --- | --- | --- | --- | --- | --- | --- | --- | --- |
|  | Ca^2+^ | Mg^2+^ | Na^+^ | K^+^ | CO_3_^2−^ | HCO_3_^−^ | SO_4_^2−^ | Cl^−^ |  |
| 864 | 5.72 | 2.02 | 7.27 | 0.38 | 0.28 | 2.68 | 4.03 | 8.4 | 3.43 |

**Supplementary Table 2: Physical and chemical properties of the experimental soil**

| **Characteristic** | **Value** |
| --- | --- |
| Texture | Sandy |
| Sand % | 91.51 |
| Silt % | 5.74 |
| Clay % | 2.75 |
| Saturation % | 23 |
| pH | 7.5 |
| Electrical conductivity (EC) (dS/m) | 2.2 |
| Organic matter (OM) % | 0.05 |
| Total N % | 0.014 |
| Available P ppm | 3.9 |
| Available K ppm | 110 |

| **Supplementary Table 3: Sequences of forward and reverse primers for real-time RT-PCR**. | | | | |
| --- | --- | --- | --- | --- |
| **Gene** | **Primers sequence** | **Amplicon length (bp)** | **GenBank accession number** | **References** |
| Diketide-CoA synthase  (DCS**)** | 5’- GTGCTGTTCATCCTGGACGAG -3’ (forward primer) | 21 | AB495006.1 | (Katsuyama et al. 2009a,b) |
|  | 5’- CAACAGCACGCCCCAGTCGA-3’  (reverse primer) | **20** |  |  |
| Curcumin synthase 1  (CURS1**)** | 5’- CATCATTGACGCCATCGAAGC -3’(forward primer) | 21 | AB495007.1 | (Katsuyama et al. 2009a,b) |
|  | 5’- TCAGCTCATCCATCACGAAGTACAC -3’(reverse primer) | 25 |  |  |
| Curcumin synthase 2  (CURS2) | 5’-TCGGGATCAAGGACTGGAACAAC-3’  (forward primer) | 23 | AB506762.1 | (Katsuyama et al. 2009a,b) |
|  | 5’-TGTTGCCGAACTCGGAGAAGAC-3’  (reverse primer) | 22 |  |  |
| Curcumin synthase 3  (CURS3) | 5’-TGGAGCCCTCCTTCGACGACC-3’  (forward primer) | 21 | AB506763.1 | (Katsuyama et al. 2009a,b) |
|  | 5’-CCCATTCCTTGATCGCCTTTTCC-3’  (reverse primer) | 23 |  |  |
| Actin | 5’-GGATATGCTCTTCCTCATGCT-3’  (forward primer) | 21 | CP002686.1  AK118354.1  AY087740.1 | (Katsuyama et al. 2009a,b) |
|  | 5’-TCTGCTGTGGTGGTGAATGA-3’  (reverse primer) | 20 |  |  |
